# Rupture of the Uterus

**Published:** 1861-10

**Authors:** Jas. S. King

**Affiliations:** Lemont, Ill., Late Resident Physician St. John’s Hospital, Cincinnati, Ohio


ARTICLE II.

### RUPTURE OF THE UTERUS.

Reported by JAS. S. KING, M. D., of Lemont, Ill.,
LATE RESIDENT PHYSICIAN ST. JOHN'S HOSPITAL, CINCINNATI, OHIO.

August 7, nine P. M.—Called to see Mrs. Mc——, a robust Irish woman, æt 34, in her second confinement. She said, that at five A. M., without any pain accompanying it, "the bag of waters had broken," since which time she had had sharp cutting pains at intervals, but still not sufficient to cause her to discontinue her usual domestic duties. During the half hour previous to my arrival, the pains had been slightly increasing. On making a vaginal examination, I found the parts of natural temperature and moist, but could not reach the os uteri. I retired, telling the nurse to call me as soon as I was needed. At half-past eleven the nurse called me, saying that Mrs. Mc——— had been having very good pains during the past hour, and that she had vomited several times. I now made another examination, and to my surprise, found the head of the child engaged in the superior strait. She continued to have pains at intervals of from five to ten minutes during the night, resting well between them; but I was surprised and perplexed to find that the head did not advance any. After a close examination, about five A. M., August 8th, I concluded that the child was dead, and that, together with the early escape of the liquor amnii, was the cause of the non-progress.

At six A. M., I left her, and returning at eight o'clock, found that there had been no change in the character of the pains, nor in her general condition; her pulse was about natural; surface moist, and she appeared in good spirits. Upon a close examination of the uterus, through the abdominal walls, I now detected an inequality of its surface, from which, together with a greater enlargement than usual, I concluded that perchance there were two children. From about nine o'clock till half-past eleven, the pains were shorter and at greater intervals, when they again increased in force and frequency, and so continued for about an hour, but without changing the position of the head, when they again diminished. I now sent for Dr. H. G. Hall, of this place, in consultation. After making an examination, and noting for a time, the character of the pains, which continued to grow less in force and frequency, Dr. H. recommended the use of ergot, in which I concurred. We administered it in the usual dose, about two P. M., and again in half an hour. The pains continued to diminish gradually until at three o'clock, (when we administered a third portion of ergot,) they had almost ceased; by four o'clock all uterine contractions had been suspended; position of child's head unchanged. She now complained of cramps and pain in right thigh, together with a feeling of uneasiness in abdomen. Pulse 90 per minute; surface of natural temperature and moist. At five o'clock we left her; returned about seven; found that there had been no return of uterine contractions, but that the trouble in abdomen and thigh was increasing. At nine o'clock we concluded to give her an opiate to quiet pain and cause her to sleep. Pulse now 100 per minute; surface of natural temperature. The opiate did not have the desired effect, the patient still continuing to complain of the "peculiar feeling" in her abdomen, and by ten o'clock she had entirely lost the use of her right leg; about the same time she became delirious; pulse now 112 per minute, and of less force; surface slightly below natural temperature. At eleven o'clock we ordered brandy to be administered. By one o'clock A. M., August 9th, her pulse had increased to 130 per minute, and feeble; surface cold; expression of countenance anxious. We now commenced giving ammonia carb., continuing also the use of the brandy, and sent a messenger to Lockport (seven miles,) for obstetrical instruments. He returned about seven A. M., but she was not now in a condition to be delivered, for notwithstanding the free use of the brandy and ammonia, she had continued to sink. Pulse now about 170 per minute, feeble and fluttering; respiration hurried and difficult; surface cold and clammy; countenance pale and ghastly; she had at intervals, since about four o'clock, vomited greenish matter. At the request of her husband, we now sent for Dr. Bacon, of Lockport, although we did not think she would live till his arrival. At eight o'clock she had a slight convulsion, and during the next half hour, she vomited several times, "coffee-ground matter," and at nine o'clock, died.

*Post Mortem*, August 9th.—At half-past one P. M., I proceeded to deliver the child, assisted by Doctors Hall and Bacon. Upon opening the abdomen, we were surprised to find a very large male child in its cavity, entirely without the uterus. After removing a quantity of sero-sanguineous fluid, and carefully removing a portion of intestine from over the child and uterus, we found that there was a rupture of the uterus, commencing about half an inch above the os uteri on the right side, thence extending up the side about two-thirds the length of the uterus, involving the contiguous peritoneum; the child had escaped through this rent into the peritoneal sac, where we found it in the following position: lying upon the right side of the abdominal cavity, arms folded over the chest, knees drawn up to the abdomen, back curved, head firmly engaged in the superior strait, with the posterior fontanelle to the right sacro-iliac synchondrosis, face to the left acetabulum. On removing the child and examining the uterus carefully, we found that its structure was not altered; it was of a purplish color; the edges of the rent jagged and uneven. The placenta, which we removed, was attached to the left side of the uterus. The os uteri was dilated just sufficient to admit of the introduction of the index finger.

---

REMARKS.—In the case under consideration, we find an absence of nearly all the symptoms of complete rupture of the uterus as laid down by our works upon obstetrics. In this case the rupture evidently took place during the time intervening between the first and second examinations, and I learn from the nurse, that she did not complain of anything unusual during that time, and that her pains were not particularly strong. There was no immediate suspension of labor-pains; no hæmorrhage whatever, from the vagina during her labor, neither was there when the liquor amnii escaped. The child's head neither receded nor advanced from the time of my second examination to the termination of the case. The collapse did not come on rapidly, but on the contrary, gradually, as will be seen by noting the symptoms. And as there was an absence of the general symptoms indicating a rupture, so was there of anything in the woman's previous history, to cause us to suspect such a calamity. Her first labor had been short, and without trouble. During her last gestation, she had enjoyed excellent health, not a symptom pointing out any disease of the uterine walls; her pelvis was not contracted, neither were the pains at the beginning of her labor severe.

